# Hsp90 inhibition and co‐incubation with pertuzumab induce internalization and degradation of trastuzumab: Implications for use of T‐DM1

**DOI:** 10.1111/jcmm.15643

**Published:** 2020-07-16

**Authors:** Marianne Skeie, Filip Nikolaysen, Ylenia Chitano, Espen Stang

**Affiliations:** ^1^ Institute of Clinical Medicine University of Oslo Oslo Norway; ^2^ Department of Pathology Oslo University Hospital Oslo Norway; ^3^ Institute for Cancer Genetics and Informatics Oslo University Hospital Oslo Norway

**Keywords:** degradation, endocytosis, HER2, Hsp90, pertuzumab, protein kinase C, T‐DM1, trastuzumab

## Abstract

The receptor tyrosine kinase HER2 is associated with a number of human malignancies and is an important therapeutic target. The antibody‐drug conjugate trastuzumab emtansine (T‐DM1; Kadcyla^®^) is recommended as a first‐line treatment for patients with HER2‐positive metastatic breast cancer. T‐DM1 combines the antibody‐induced effects of the anti‐HER2 antibody trastuzumab (Herceptin^®^) with the cytotoxic effect of the tubulin inhibitor mertansine (DM1). For DM1 to have effect, the T‐DM1‐HER2 complex has to be internalized and the trastuzumab part of T‐DM1 has to be degraded. HER2 is, however, considered endocytosis‐resistant. As a result of this, trastuzumab is only internalized to a highly limited extent, and if internalized, it is rapidly recycled. The exact reasons for the endocytosis resistance of HER2 are not clear, but it is stabilized by heat‐shock protein 90 (Hsp90) and Hsp90 inhibitors induce internalization and degradation of HER2. HER2 can also be internalized upon activation of protein kinase C, and contrary to trastuzumab alone, the combination of two or more anti‐HER2 antibodies can induce efficient internalization and degradation of HER2. With intention to find ways to improve the action of T‐DM1, we investigated how different ways of inducing HER2 internalization leads to degradation of trastuzumab. The results show that although both Hsp90 inhibition and activation of protein kinase C induce internalization of trastuzumab, only Hsp90 inhibition induces degradation. Furthermore, we find that antibody internalization and degradation are increased when trastuzumab is combined with the clinically approved anti‐HER2 antibody pertuzumab (Perjeta^®^).

## 1 INTRODUCTION

HER2/ErbB2 is associated with several human malignancies and is an important therapeutic target [reviewed in^1^]. HER2 has no known ligand, but is the preferred heterodimerization partner. Furthermore, HER2 is endocytosis‐deficient and retains its partner at the plasma membrane. All this contribute to a high oncogenic potential [reviewed in^2^].

Monoclonal antibodies (mAbs) are important in cancer treatment. Antibody‐dependent cellular cytotoxicity (ADCC) is important, but mAbs may also inhibit ligand binding and receptor dimerization, and/or induce receptor internalization and degradation, and as such inhibit downstream signalling. The anti‐HER2 antibody trastuzumab (Herceptin^®^) was one of the first mAbs approved. Trastuzumab stimulates NK cell–induced lysis of HER2‐overexpressing cells.^1^ Whether trastuzumab induces endocytosis of HER2 is discussed. Most studies conclude that internalization of trastuzumab‐HER2 complexes is highly limited, and if internalized, they are recycled and not degraded.^2^ The anti‐HER2 antibody pertuzumab (Perjeta^®^) is approved for use in combination with trastuzumab and docetaxel.^1^ The approval was based on the phase III study CLEOPATRA, which showed that the combination significantly improved survival of patients with HER2‐positive metastatic breast cancer (MBC).^3^

As a development of mAbs, antibody‐drug conjugates (ADCs) have become important cancer treatment tools. Trastuzumab emtansine (T‐DM1; Kadcyla^®^), trastuzumab linked to the microtubule inhibitor emtansine (DM1), was one of the first ADCs approved. Based on the phase III study EMILIA, T‐DM1 was approved for HER2‐positive, late‐stage MBC. Based on the phase III study KATHERINE, T‐DM1 was recently approved also for adjuvant treatment of a subgroup of patients with HER2‐positive early breast cancer. Although T‐DM1 is promising, other trials concluded that its efficiency is unclear [reviewed in^4^, ^5^]. Resistance is one problem. DM1 is attached by a non‐cleavable linker, and its release depends on internalization and degradation of the antibody. Inhibited internalization or reduced lysosomal activity can thus cause resistance.^6^, ^7^

Why HER2 is endocytosis‐deficient is unclear, but HER2 is stabilized by Hsp90, and Hsp90 inhibitors induce endocytosis and degradation of HER2.^2^ Activation of protein kinase C (PKC) also induces HER2 internalization, but unlike Hsp90 inhibition, it does not induce degradation.^8^ We recently showed that Hsp90 inhibition and PKC activation also induce internalization of trastuzumab. However, although trastuzumab upon Hsp90 inhibition was routed to late endosomes, it was retained in recycling compartments upon PKC activation.^8^ Also, the combination of two or more antibodies, recognizing different HER2 epitopes, can induce efficient internalization and degradation of HER2.^9^, ^10^, ^11^

Treatment modules which increase trastuzumab internalization and degradation may reduce the T‐DM1 dose needed and as such reduce its adverse effects. Using trastuzumab as model, we now studied under which conditions it is degraded.

## 2 METHODS

SK‐BR‐3 cells were studied using immunoblotting, flow cytometry and immuno‐electron microscopy (see Appendix S1).

## 3 RESULTS AND DISCUSSION

### 3.1 Hsp90 inhibition, but not PKC activation, causes degradation of trastuzumab

**Figure 1:**
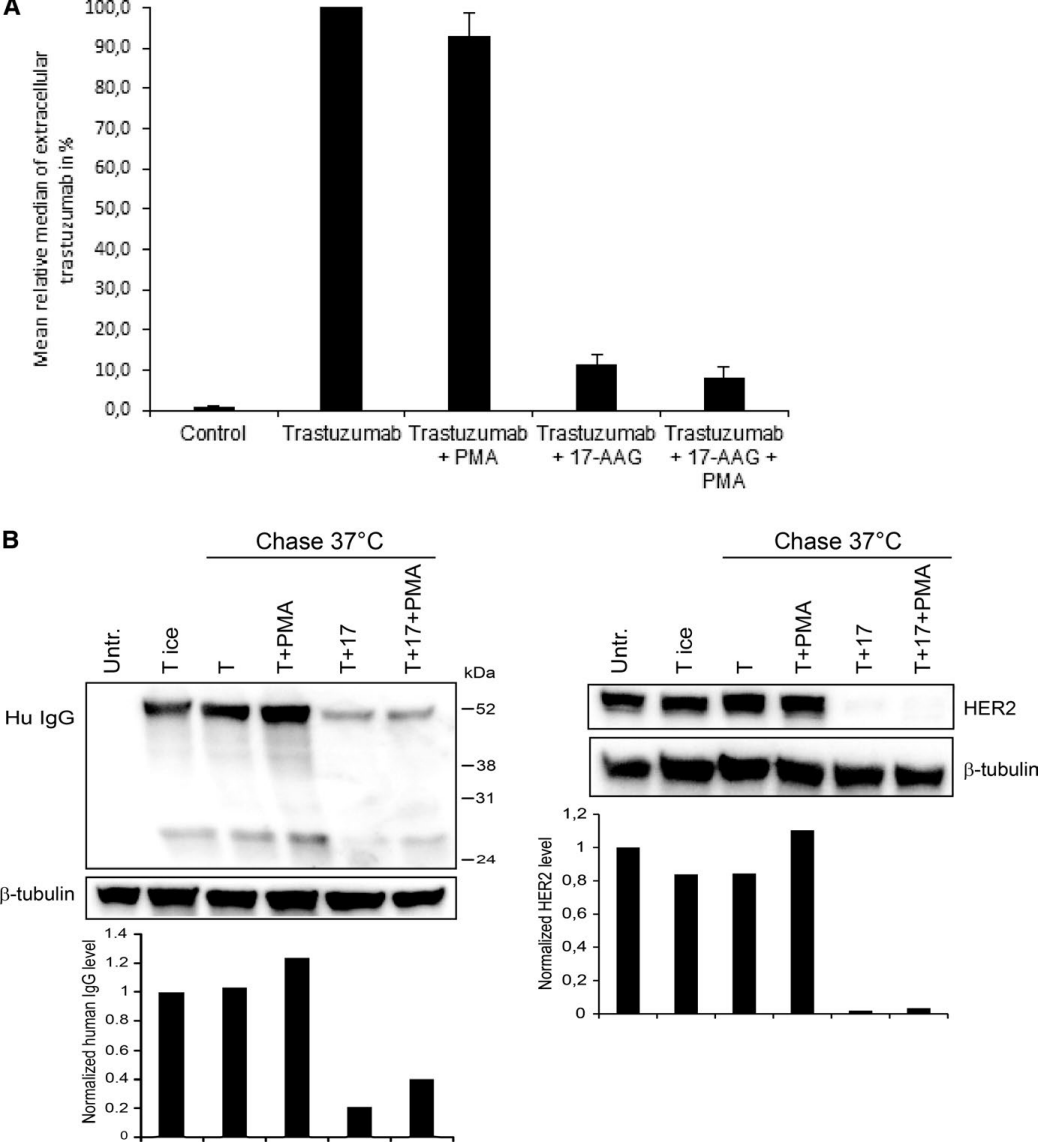
Hsp90 inhibition, but not PKC activation, induces degradation of trastuzumab. A, SK‐BR‐3 cells were incubated with trastuzumab on ice for 30 min followed by chase at 37°C for 4 h in antibody‐free medium with or without the Hsp90 inhibitor 17‐AAG and/or the PKC activator PMA. Cells were fixed and stained for flow cytometry using Alexa Fluor 488‐conjugated goat anti‐human IgG. Data from three independent experiments were included. The measured level for plasma membrane–localized trastuzumab (extracellular trastuzumab) in cells not treated with 17‐AAG or PMA was in each experiment set to 100%. B, SK‐BR‐3 cells were left untreated or incubated with trastuzumab (T) on ice for 30 min before chase for 6 h at 37°C in antibody‐free medium with or without PMA and/or 17‐AAG. Cells were lysed and subjected to immunoblotting using antibodies to human IgG (Hu IgG), HER2 and β‐tubulin (loading control). Blots are representative of the three experiments done. Net luminescence of the bands corresponding to human IgG or HER2 was quantified, normalized to tubulin and plotted relative to Hu IgG in cells with trastuzumab on ice (T ice) or HER2 in control (Untr.) cells, respectively

Our previous study^8^ indicated that trastuzumab is not degraded unless HER2 internalization is induced by treatments like Hsp90 inhibition. To study this further, SK‐BR‐3 cells were incubated with trastuzumab on ice followed by chase at 37°C in antibody‐free medium with or without the Hsp90 inhibitor 17‐AAG and/or the PKC activator PMA. Flow cytometry showed that 17‐AAG induced a strong decrease in plasma membrane–localized human IgG (Figure 1A). Also, PMA reduced this, but far less efficiently. To study trastuzumab degradation, cell lysates were immunoblotted using antibodies to human IgG. The IgG level did not decrease when cells were chased in normal medium (Figure 1B), but decreased significantly when 17‐AAG was added. PMA did not induce any decrease. In line with this, 17‐AAG, but not PMA, induced efficient down‐regulation of HER2. When 17‐AAG and PMA were combined, both trastuzumab and HER2 were efficiently down‐regulated.

A decrease in cell‐associated trastuzumab may represent antibody dissociation and not necessarily degradation. However, the minimal decrease upon chase in normal medium indicates that trastuzumab is tightly bound. Using confocal microscopy, we previously showed that trastuzumab remains at the plasma membrane under normal conditions, but becomes internalized upon treatment with 17‐AAG or PMA.^8^ To characterize this at high resolution, we studied the localization of trastuzumab by immuno‐electron microscopy (immuno‐EM). Upon chase in normal medium, labelling for human IgG was almost completely restricted to the plasma membrane. Only minimal labelling was found in endosome‐like compartments (Figure S1). However, when cells were chased with 17‐AAG, strong labelling was found in early endosome‐like compartments and multivesicular bodies (MVBs), most likely representing late endosomes. In MVBs, labelling was concentrated on internal vesicles, indicating transport to lysosomes for degradation. Some labelling was also found in dense lysosome‐like compartments. In cells chased with PMA, internalization was less efficient. However, in line with our study of HER2 localization,^8^ human IgG was found in complex tubulovesicular/cisternal compartments, supporting that PKC activation induces internalization and retention, but not degradation, of HER2‐trastuzumab complexes.

### 3.2 Combining trastuzumab with pertuzumab increases antibody internalization and degradation

**Figure 2:**
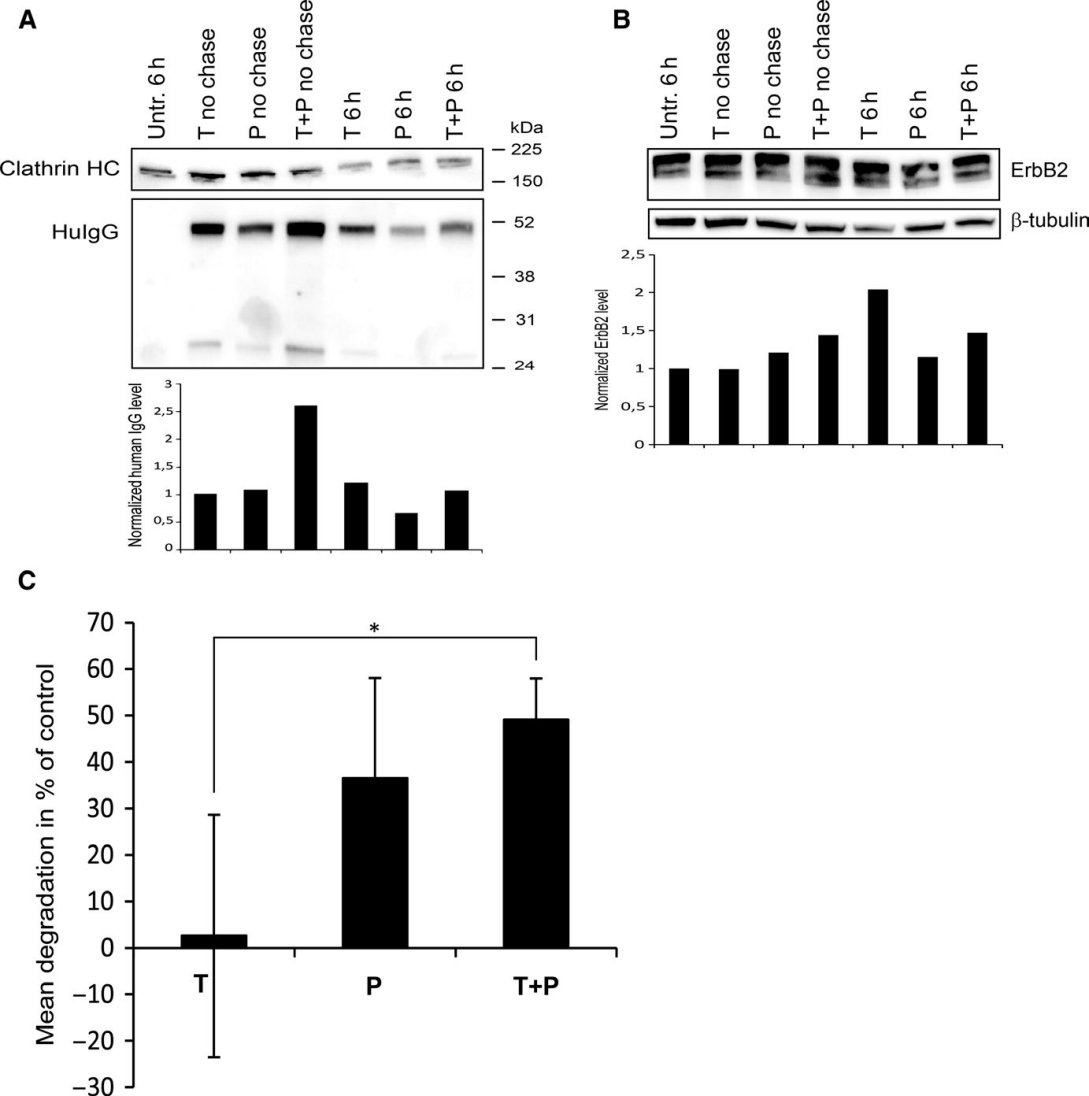
The combination of trastuzumab with pertuzumab induces significant degradation of human IgG. SK‐BR‐3 cells were incubated with trastuzumab or pertuzumab alone and in combination for 30 min on ice before chase at 37°C in antibody‐free medium for 6 h. Cells were lysed and subjected to immunoblotting using antibodies to either (A) human IgG and clathrin heavy chain (loading control), or (B) HER2 and β‐tubulin (loading control). Blots are representative of the three experiments done. Net luminescence of the bands corresponding to human IgG or HER2 was quantified, normalized to clathrin heavy chain or β‐tubulin and plotted relative to Hu IgG in cells with trastuzumab on ice (T no chase) or HER2 in control (Untr.) cells, respectively. (C) Degradation of human IgG upon chase at 37°C as compared to control (incubation on ice). The figure shows mean increase in degradation as quantified from three independent experiments

Alternative ways to induce T‐DM1 internalization are, however, important. As the combination of antibodies recognizing different HER2 domains increases internalization and inhibits recycling of antibody‐HER2 complexes,^2^, ^10^, ^11^ we studied the localization and degradation of human IgG in cells exposed to trastuzumab or pertuzumab alone, or in combination. The cells were incubated with antibodies on ice before chase in antibody‐free medium at 37°C. Immuno‐EM showed strong labelling for human IgG at the plasma membrane under all conditions, but cells exposed to the antibody combination additionally showed labelling in endosome‐like compartments (Figure S2). Importantly, immunoblotting clearly shows that antibody degradation was increased when the two antibodies were combined (Figure 2). In immuno‐EM and immunoblotting, we used anti‐human IgG antibodies which do not differentiate between trastuzumab and pertuzumab, but it is likely that both antibodies are degraded to a similar degree.

This indicates that the effect of T‐DM1 may increase if combined with pertuzumab or other anti‐HER2 antibodies which do not compete with trastuzumab. Supporting this, Phillips et al^12^ found that T‐DM1 and pertuzumab in combination induced increased apoptosis compared to each reagent alone and the combination resulted in enhanced antitumour activity in mouse models. Likewise, a phase II trial in patients with HER2‐positive MBC showed that the combination had no unexpected toxicities and the efficacy appeared promising.^13^ The phase III trial MARIANNE showed that T‐DM1 and T‐DM1 + pertuzumab were non‐inferior, but not superior, to trastuzumab + taxane. However, the study showed no clear increase in overall survival with pertuzumab and it was concluded to recommend T‐DM1 as a first‐line treatment for patients with HER2‐positive MBC.^14^ This does, however, not rule out that the therapeutic effect of T‐DM1 can increase when combined with other anti‐HER2 antibodies.

## 4 CONCLUSIONS

Our data strongly indicate that the effect of T‐DM1 will increase if combined with Hsp90 inhibitors or anti‐HER2 antibodies. In support of this, 17‐AAG has been shown to increase the internalization and effect of a similar trastuzumab‐drug conjugate, Trast‐NG/DOX, both in vitro and in vivo. Importantly, the effect was strongly increased even at sub‐therapeutic doses of 17‐AAG.^15^ This could suggest that T‐DM1 can be used in combination with Hsp90 inhibitors at doses with acceptable adverse effects. Likewise, several anti‐HER2 antibodies, including ADCs,^4^, ^5^, ^15^ are in clinical trials. It will be important to test T‐DM1 in combination with one or more of these.

## CONFLICT OF INTEREST

The authors declare that they have no conflicts of interest with the contents of this article.

## AUTHOR CONTRIBUTIONS

**Marianne Skeie:** Data curation (equal); Investigation (equal); Methodology (supporting); Visualization (supporting); Writing‐original draft (supporting); Writing–review & editing (supporting). **Filip Nikolaysen:** Data curation (supporting); Investigation (supporting). **Ylenia Chitano:** Data curation (supporting); Investigation (supporting); Visualization (supporting). **Espen Stang:** Conceptualization (lead); Data curation (equal); Funding acquisition (lead); Investigation (equal); Methodology (equal); Project administration (lead); Supervision (lead); Validation (lead); Visualization (equal); Writing–original draft (lead); Writing–review & editing (lead).

## Supporting information

Appendix S1

## Data Availability

The data that support the findings of this study are available from the corresponding author upon reasonable request.
