# Supplementary material for: Hsp90 inhibition and co‐incubation with pertuzumab induce internalization and degradation of trastuzumab: Implications for use of T‐DM1
Source: J Cell Mol Med. 2020 Jul 16;24(17):10258–62. doi: 10.1111/jcmm.15643 (PMC7520337; doi:10.1111/jcmm.15643)
Supplement: Supplementary file 1 — Appendix S1 [file JCMM-24-10258-s001.pdf]

Supplementary information

MATERIALS AND METHODS

Antibodies and materials

The following antibodies were used: Alexa Fluor 488-conjugated goat anti-human IgG (Thermo Fisher Scientific), rabbit anti-HER2 (Tyr 1248) (Cell Signaling Technology, Inc.), rabbit anti-β-tubulin (Abcam), mouse anti-clathrin heavy chain (BD Biosciences), peroxidase-conjugated donkey anti-rabbit and anti-mouse, and rabbit anti-human IgG (Jackson ImmunoResearch Laboratories), trastuzumab (Herceptin®) and pertuzumab (Perjeta®) (Roche Pharma) and rabbit anti-mouse IgG (Cappel Research Reagents ICN Biochemicals). 17-AAG was from Tocris Bioscience (BioTechne Ltd.). Unless noted, all other materials and chemicals were from Sigma-Aldrich.

Cell Culture and Treatment

SK-BR-3 cells were grown in DMEM containing Ultraglutamine I, 4.5 g/l Glucose (Lonza), 50 units/ml Potassium Penicillin, 50 µg/ml Streptomycin Sulfate and 15% (v/v) fetal bovine serum. 3 µM of 17-AAG and/or 100 nM of PMA were routinely used. Trastuzumab and pertuzumab, each 25µg/ml, were diluted in MEM (Thermo Fisher Scientific) containing 0.1% BSA.

Flow Cytometry

Cells were prepared and flow cytometry was performed as previously described [1]. Alexa 488-conjugated anti-human IgG was used to detect trastuzumab bound to plasma membrane-localized HER2.

Immunoblotting

Cell lysates were prepared and immunoblotting was performed basically as described in [1]. Immobilon Forte Western HRP substrate (Merck Millipore) was used for protein detection.

Immuno-electron microscopy

Cells were prepared for cryo immuno-electron microscopy as previously described [1]. Labeling for human IgG was done using rabbit anti-human IgG followed by protein A gold (G. Posthuma, Utrecht, The Netherlands). Adobe Photoshop was used for image processing.

Statistical Data Analysis

Results from at least three independent experiments were statistically analysed using Microsoft Excel. Mean ± SD was calculated, and significance determined by unpaired t test type 2 (variances of subpopulations were equal by F-test). Significance is denoted as *, $P<0.05$.

References

1. Dietrich, M., et al., *Protein kinase C mediated internalization of ErbB2 is independent of clathrin, ubiquitination and Hsp90 dissociation.* Exp Cell Res, 2018. **371**(1): p. 139-150.

Supplementary figures

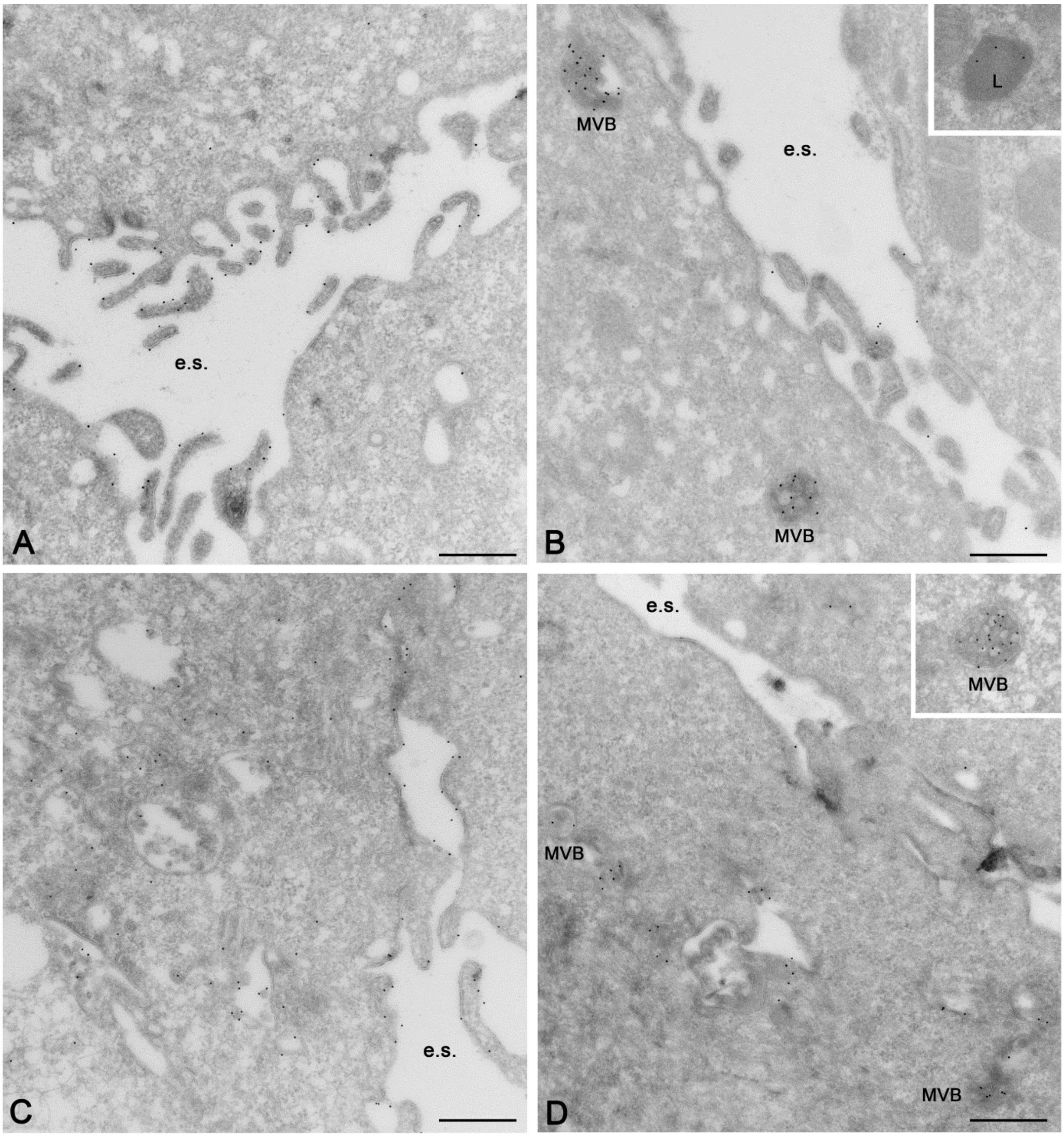

Supplementary figure 1: Only Hsp90 inhibition induces efficient internalization of trastuzumab to multivesicular bodies. SK-BR-3 cells were incubated with trastuzumab on ice for 30 min before chase for 3 h at 37°C in antibody-free medium without additives (A), or medium containing 17-AAG (B), PMA (C) or 17-AAG and PMA (D). The sections were labeled using antibodies to human IgG followed by 10 nm protein A gold. MVB: multivesicular body; L: lysosome-like compartment; e.s.: extracellular space. Scale bars: 500 nm.

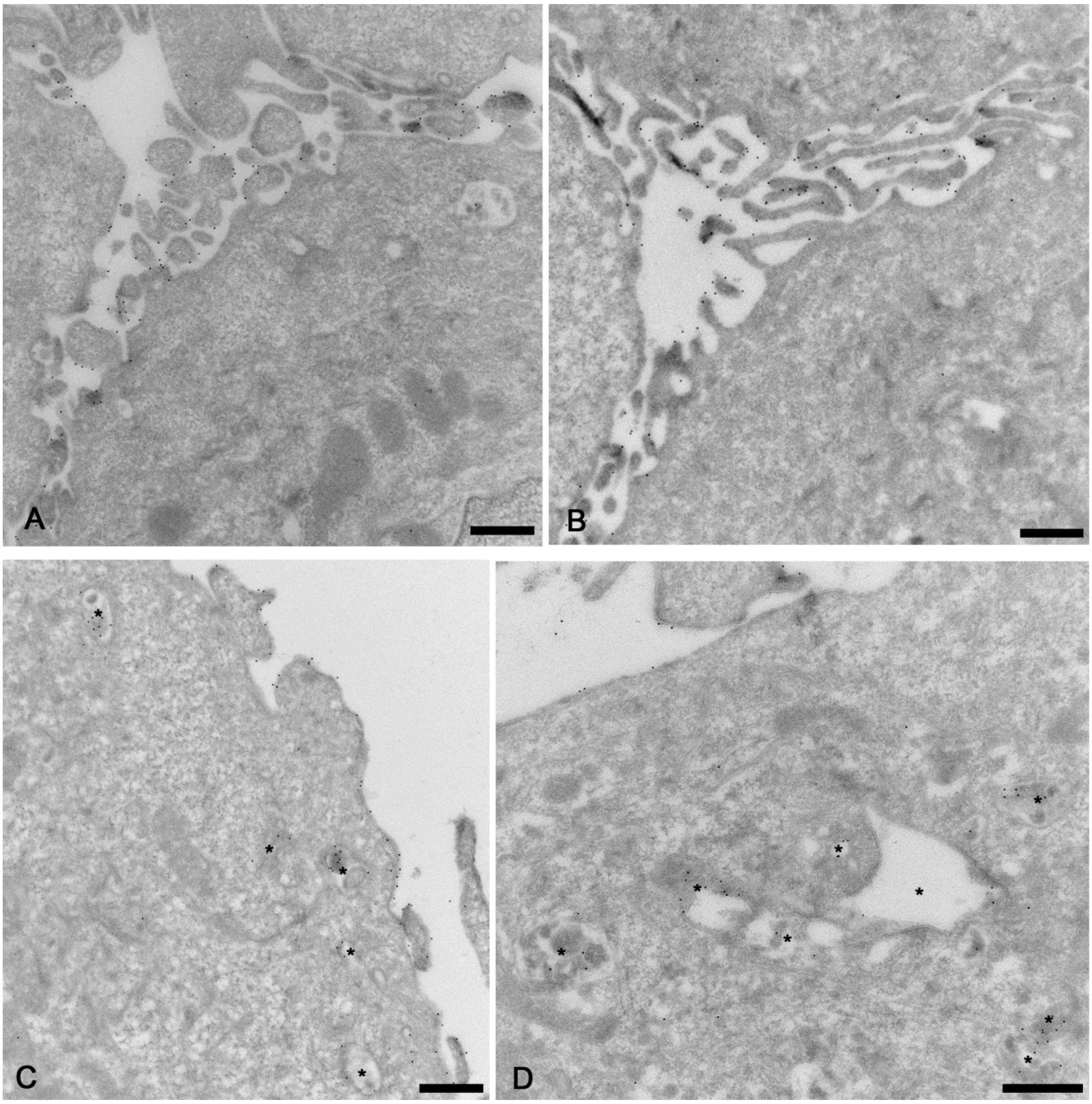

Supplementary figure 2: Trastuzumab and pertuzumab must be combined to become internalized. SK-BR-3 cells were incubated with either trastuzumab (A), pertuzumab (B) or trastuzumab and pertuzumab in combination (C,D) on ice for 30 min before chase at 37°C in antibody-free medium for 4 h. The sections were labeled using antibodies to human IgG followed by 10 nm protein A gold. * indicates endosomal compartments positive for human IgG. Scale bars: 500 nm.
